# Novel Serratus Posterior Superior Intercostal Plane Block Provided Satisfactory Analgesia after Breast Cancer Surgery: Two Case Reports

**DOI:** 10.4274/TJAR.2024.231431

**Published:** 2024-02-28

**Authors:** Gökçen Kültüroğlu, Savaş Altınsoy, Yusuf Özgüner, Cem Koray Çataroğlu

**Affiliations:** 1 Etlik City Hospital, Clinic of Anaesthesiology and Reanimation, Ankara, Turkey

**Keywords:** Analgesia, breast cancer nerve block, pain, perioperative care, regional anaesthesia

## Abstract

The serratus posterior superior intercostal plane (SPSIP) block is a novel technique recently described for thoracic analgesia. This study presents two cases using this technique for postoperative pain after mastectomy with axillary lymph node dissection. The SPSIP block was administered to the patients in the preoperative period as part of multimodal analgesia, and postoperative pain was monitored using the numeric rating scale (NRS). In both patients, the NRS pain scores were below 3/10. SPSIP provided adequate postoperative analgesia in these cases without the need for any opioid agents. Thus, an SPSIP block can be a valuable treatment option for postoperative pain after breast surgery.

**Main Points**

• The serratus posterior superior intercostal plane block is a newly defined superior trunk block.

• This block, which is applied from the medial side of the scapula, provides analgesia for the ipsilateral hemithorax and axillary region.

• This block may be an effective alternative analgesic method for breast surgeries that include axillary lymph node dissection.

## Introduction

The serratus posterior superior intercostal plane (SPSIP) block is a novel regional technique for thoracic analgesia described by Tulgar et al.^1^ in 2023. Their cadaveric study demonstrated that the dye spread from the 7^th^ cervical vertebra to the 7^th^ thoracic vertebra following the injection of 30 mL of methylene blue into the fascial plane between the serratus posterior superior and intercostal muscles. In patients with myofascial pain syndrome, the block provided analgesia for both the anterior and posterior hemithorax.^1^

This study included two patients who received SPSIP blocks for elective unilateral-modified radical mastectomy with axillary lymph node dissection.

## Case Presentation

**Figure 1:**
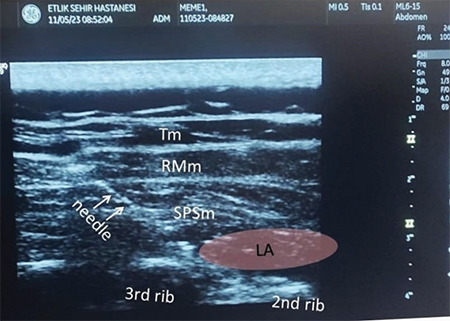
Local anaesthetic infiltration into the plane between the serratus posterior superior muscle and intercostal muscle. Tm, trapezius muscle; RMm, rhomboid major muscle; SPSm, serratus posterior superior muscle; LA, local anaesthetic.

The first patient was a 54-year-old female, and her ASA physical status was II because of hypothyroidism. The second patient was a 46-year-old female with an ASA physical status of I. Both patients signed informed consent forms for inclusion in the study. After standard monitoring and intravenous (IV) access were provided to the patients, SPSIP block was performed in the sitting position under sterile conditions as previously described.^1^ To help lateralize the scapula, the patient was directed to hold their opposite shoulder using the hand on the surgical or blocked side. A linear ultrasound probe was positioned along the sagittal plane to the medial border of the scapula. Upon identification of the second and third ribs, trapezius, rhomboid, serratus posterior superior, and intercostal muscles, as well as the pleura, a 30 mL injection of 0.25% bupivacaine was administered into the interfascial plane between the serratus posterior superior and intercostal muscles. The diffusion of a local anaesthetic to the cephalad and caudad in the interfascial plane was visualized using ultrasonography (Figure 1). Routine anaesthesia induction using intravenous lidocaine, fentanyl, propofol, and vecuronium bromide was performed. At the end of the surgery, 50 mg of dexketoprofen, 4 mg of ondansetron, and 40 mg of pantoprazole were intravenously administered. The total surgical duration was 100 and 130 min. The numeric rating scale (NRS) assessed postoperative pain at minutes 15 and 30 and 1, 2, 6, 12, and 24 h after surgery. The patients received 2 IV doses of acetaminophen (1 gr/dose) at 8 h intervals for postoperative analgesia. The NRS pain scores in both patients were 3/10 in the first 24 h. The patients did not require opioid analgesics, and no perioperative complications developed. Moreover, motor block was not observed in either patient.

## Discussion

In breast cancer surgery, regional techniques are an important part of postoperative pain management for chronic pain prevention and patient safety.^2,3^ The breast is innervated by several branches of the thoracic intercostal and supraclavicular nerves; accordingly, regional techniques can be applied to many planes. Studies have shown that postoperative pain and opioid consumption are reduced in all procedures, including the pectoral nerve, paravertebral nerve, serratus anterior plane, erector spinae plane, and rhomboid intercostal blocks.^4,5^ The SPSIP block can be investigated as a new plane block in breast surgery.

After the SPSIP block was defined and successful results for myofascial pain syndrome were obtained, a case report in which an SPSIP block was applied for acute postoperative pain was published.^6^ It reported that adequate analgesia had been obtained for postoperative acute pain after breast surgery, similar to the results of this study. Because the breast is innervated by an extensive nerve network and the breast pain mechanism is complex, breast surgery requires multimodal analgesia, including regional methods.^7^ This study applied preoperative SPSIP blocks and administered a nonsteroidal anti-inflammatory drug and two doses of acetaminophen to the patients postoperatively. Using this protocol, patient pain was controlled in the first 24 h after surgery without opioids.

Rhomboid block is performed at the inferior-medial border of the scapula and is a reliable peripheral block that provides analgesia between T2-T9 dermatomes.^8^ There are many publications reporting that it reduces patient pain scores and opioid consumption after breast surgery.^9,10^ However, there is also an article reporting acceptable pain in the axilla after axillary lymph node dissection.^11^ Because the SPSIP block is more superiorly located than the rhomboid intercostal plane block, it may be more effective in analgesia of the upper thoracic dermatomes. However, prospective randomized studies are required to clarify this possibility.

The effectiveness of fascial plan blocks is multifactorial: local anaesthetic type, concentration and volume, patient age, use of muscle relaxants, and surgical injury. In a report describing the SPSIP block, 20 mL, 30 mL, and 40 mL 0.25% bupivacaine were used for 5 patients and they obtained similar sensory block findings.^1^ In a recently published study, Avci et al.^12^ performed SPSIP with 30 mL 0.25% bupivacaine for thoracoscopic surgery and demonstrated effective analgesia. To provide effective analgesia and avoid toxic effects, we preferred to use 30 mL 0.25% bupivacaine for the ship block.

It is too early to discuss the advantages and disadvantages of the SPSIP block for breast surgery because high-quality randomized controlled studies or meta-analyses have not been published. However, two main advantages can be identified; the injection site is away from the surgical area, and the block provides analgesia for the entire axillary area. Nonetheless, patients may feel numbness in the ipsilateral neck region, which may cause anxiety. This problem can be solved by providing the patient with detailed information during the pre-operative period.

## Conclusion

Although the SPSIP block has had positive results in a series of patients with chronic pain, its use for acute pain management after breast cancer surgery is a very new approach. In this case study, SPSIP blocks provided adequate postoperative analgesia during the first 24 h after breast surgery. However, this approach should be further supported through randomized controlled trials.
